# Fertility-Sparing Approach in Women Affected by Stage I and Low-Grade Endometrial Carcinoma: An Updated Overview

**DOI:** 10.3390/ijms222111825

**Published:** 2021-10-31

**Authors:** Giuseppe Gullo, Andrea Etrusco, Gaspare Cucinella, Antonino Perino, Vito Chiantera, Antonio Simone Laganà, Rossella Tomaiuolo, Amerigo Vitagliano, Pierluigi Giampaolino, Marco Noventa, Alessandra Andrisani, Giovanni Buzzaccarini

**Affiliations:** 1 Department of Obstetrics and Gynecology, Villa Sofia Cervello Hospital, IVF UNIT, University of Palermo, 90146 Palermo, Italy; gullogiuseppe@libero.it (G.G.); gaspare.cucinella1@gmail.com (G.C.); antonio.perino@unipa.it (A.P.); 2 Department of Obstetrics and Gynecology, Villa Sofia Cervello Hospital, University of Palermo, 90146 Palermo, Italy; etruscoandrea@gmail.com; 3 Department of Gynecology Oncology, University of Palermo, 90146 Palermo, Italy; vito.chiantera@gmail.it; 4 Department of Obstetrics and Gynecology, “Filippo Del Ponte” Hospital, University of Insubria, 21100 Varese, Italy; antoniosimone.lagana@uninsubria.it; 5 Vita-Salute San Raffaele University, 20132 Milan, Italy; tomaiuolo.rossella@hsr.it; 6 Department of Women’s and Children’s Health, Gynaecological Clinic, University of Padova, 35128 Padova, Italy; amerigo.vitagliano@gmail.com (A.V.); marco.noventa@unipd.it (M.N.); alessandra.andrisani@unipd.it (A.A.); 7 Department of Public Health, University Federico II of Naples, 80138 Naples, Italy; pgiampaolino@gmail.com

**Keywords:** endometrial cancer, fertility sparing, fertility preservation, ART, infertility, progestin, IUD, metformin

## Abstract

Endometrial cancer (EC) is a deleterious condition which strongly affects a woman’s quality of life. Although aggressive interventions should be considered to treat high-grade EC, a conservative approach should be taken into consideration for women wishing to conceive. In this scenario, we present an overview about the EC fertility-sparing approach state of art. Type I EC at low stage is the only histological type which can be addressed with a fertility-sparing approach. Moreover, no myometrium and/or adnexal invasion should be seen, and lymph-vascular space should not be involved. Regarding the pharmaceutical target, progestins, in particular medroxyprogesterone acetate (MPA) or megestrol acetate (MA), are the most employed agent in conservative treatment of early-stage EC. The metformin usage and hysteroscopic assessment is still under debate, despite promising results. Particularly strict and imperious attention should be given to the follow-up and psychological wellbeing of women, especially because of the double detrimental impairment: both EC and EC-related infertility consequences.

## 1. Introduction

Endometrial cancer (EC) is the fifth most common cancer in women worldwide and the most common malignancy of the female genital tract in developed countries [1,2]. Most women are diagnosed postmenopausal, and although rare in young patients, 14–25% of patients affected by EC are premenopausal and 5% of them are under 40 years of age, and for many of them, the desire of motherhood has not yet been satisfied [3]. Risk factors for EC include nulliparity, early menarche, late age at menopause, age >55 years, ovarian disease such as PCOS, therapy with tamoxifen, chronic liver disease, and obesity [4,5]. Table 1 shows the risk factors detected for endometrial cancer, ovarian cancer, and cervical cancer etiopathogenesis, according to the strength of association.

Most patients with EC typically show high BMI or are obese, and they also have often some components of metabolic syndrome, such as hypertension and/or diabetes. EC can be distinguished in two main clinic and pathological/molecular types: I and II. 

Type I is the endometrioid type (EEC) and is the most common. It tends to develop in a younger cohort of women and includes the perimenopausal group [6]. This type of cancer is often estrogen dependent, minimally invasive, and low grade. This is the reason why they are better in terms of prognosis. 

Type II endometrial cancer is typically found in older patients. This cancer is not estrogen dependent, usually is aggressive, and of high stage and grade. It presents a non-endometrioid histology, deeply invades the myometrium and has the worst prognosis [7].

Most endometrial cancer cases are sporadic and only 10% of them are considered familiar. Particularly, patients with EC at a younger age may have a genetic etiology. Usually, EC is associated to Lynch syndrome [8]. Diagnosis is mainly performed by the execution of hysteroscopy (HSC) with endometrial biopsy [9]. However, moderate agreement is still found when comparing HSC to post-operatory histologic examination [10]. To sum up, among curettage and hysteroscopic guided biopsy, the hysteroscopy resection reduces the risk of underdiagnosed endometrial cancer [11]. Imaging performed by MRI or transvaginal ultrasounds by experts, are useful to detect possible myometrial invasion and exclude synchronous ovarian cancer or ovarian metastasis [12]. Standard treatment for EC, which includes total hysterectomy and bilateral salpingo-oophorectomy (TH-BSO), has excellent outcomes in terms of survival [13]. However, such operation leads to permanent loss of fertility, and for women who want to preserve their fertility, conservative methods must be investigated. A diagnosis of Lynch Syndrome and the associated risks for EC and ovarian cancer might impact a patient’s decision about fertility-sparing treatment [8,14]. Fertility-sparing methods include hormonal therapy with oral progestins and/or levonorgestrel-releasing IUD (LNG-IUD), surgical treatment by hysteroscopy and, only in recent times, also the combined use of metformin may evaluate. After complete remission, women must be encouraged to conceive. Even if some conservative methods have been proposed to preserve female fertility of patients with low grade and low stage of EC, the optimal management of these patients is still unknown [15].

## 2. Discussion

### 2.1. Target Patients for Fertility-Sparing Treatment

When considering a conservative management approach, the characteristics of the disease should be evaluated to select the therapeutic approach that perfectly fits for the patients. Fertility sparing could be considered for patients who have interest to preserve their fertility and plan to conceive as soon as possible after remission, with no medical contraindication to the medical treatment and favorable histopathological characteristics of the cancer. However, patients should be informed that the fertility-sparing option is not a standard management and treatment for EC, and they must be encouraged to undergo a TH-BSO after pregnancy [2]. Traditionally, G1 and EEC are the most suitable kind of malignancy for fertility-sparing treatment. On the contrary, EC type II usually is deeply invasive and poorly differentiated, and these patients are ineligible for conservative treatments.

Thus, the ideal candidates for fertility-sparing treatment have been suggested to be young women with G1, no myometrium and/or adnexal invasion, and without lymph-vascular space involvement [2,16]. These patients are more likely to present estrogen and progesterone receptors, with positive tumors and progestin treatment results in encouraging outcomes. Although low grade of the disease and absence of myometrial invasion is a requirement that would be better to have, few studies conducted on patients with advanced EC and with unsatisfied desire for motherhood assessed the outcomes of fertility sparing for these kinds of patients [17].

Chae et al. [18] reported that higher grade is associated with pregnancy failure after fertility-sparing treatment. Park et al. [17] reported outcomes of fertility-sparing treatment for grade 2–3 EC with or without myometrial invasion. The rates of complete response to fertility-sparing treatment were lower and lower as the severity of the disease increased. Some guidelines suggested that myometrial invasion should be considered as contraindication for fertility-sparing treatment [19]. 

The Japan Society of Gynecologic Oncology, the European Society of Gynecological Oncology and the Korean Society of Gynecologic Oncology have established that fertility-sparing treatment should be considered only for patients with grade 1 EEC confined to the endometrium [20]. The British Gynecological Cancer Society suggested that fertility- sparing treatment may be safe only for a short term and in patients with grade 1 ECC and, at most, a superficial myometrial invasion [21]. 

MRI is known to be the best method and offers the highest efficacy to determine the myometrial invasion [22]. According to Federation International Gynecologic and Obstetrics (FIGO), myometrial invasion is the most important prognostic factor for lymph-node invasion in patients with EC [23]. In presence of lymph-node metastasis, mortality is high and fertility sparing is not safe and should be avoided [24].

### 2.2. Hormonal Approach for Fertility Sparing

Although literature exists regarding medical treatment for fertility sparing, it is necessary to specify that there is no consensus on which agent, dose, or duration of treatment is most effective. Generally, in accord with the present published literature, progestin, in particular medroxyprogesterone acetate (MPA) or megestrol acetate (MA) at 400–600 mg/daily and 160–320 mg/daily, respectively, tends to be the most employed agent in conservative treatment of early-stage EC [25]. A complete response of treatment after therapy with progestin occurs in about 50% of patients on average, with around 5.5 months of continuous use [1,26,27]. Despite a positive response, 1/5 of patients treated only with progestin reported a recurrence of the disease [26]. Yamazawa et al. [27] reported that recurrence takes place after 23+/− months. Additionally, relapse was documented [25] for a small percentual of patients after their initial total response. Park et al. [28], in a retrospective study, demonstrated the safety of fertility-sparing treatment with MPA or MA for EEC; in fact, of the 148 patients recruited for the study, nobody shows clinical progression. The study also concluded that MPA was associated with a lower risk of recurrence than MA. However, MA has been linked to higher remission probabilities compared to MPA [29]. In patients who receive long-term oral administration of progesterone, close monitoring and endometrial sampling is mandatory every 3–6 months [1], and if there is a progression of EC and/or a continuous presence of EC after 6–12 months of progestin therapy, TH +/− SBO is highly recommended [1,2]. Unluckily, long-term oral administration of progestin may cause side effects in patients including thrombophlebitis, pulmonary emboli, hypertension, weight gain, depression, headache and abdominal cramps, and for this reason, complete compliance by the patient is difficult [30,31,32,33]. That is the reason why continuous conservative treatment may also include an IUD containing levonorgestrel (LNG), alone or in combination with MPA/MA or gonadotropin-releasing hormone agonist (GnRHa) [1,25,32,34], and also the reason why more and more women choose to accept this option for conservative management of endometrial hyperplasia and EC [32,33]. The LNG-IUD can bypass the issues with patient noncompliance due to progestin adverse reactions as well as increase local effectiveness. Yuk et al. [35], in a recent meta-analysis, compared the LNG-IUD to oral administration of MPA, finding that regression rate for both was similar overall. Pal et al. [36] demonstrated that therapy with LNG-IUD in patients with atypical hyperplasia or early-stage EC leads in a return to a normal histology in most cases. However, Wei et al. [2] demonstrated that therapy with LNG-IUD alone became worse with pregnancy outcomes. Kim et al. [37] evaluated the efficacy of LNG-IUD with oral administration of MPA 500 mg/daily, the complete remission was 87.5%, and around 12% continued to have successful pregnancies, concluding that combined treatment is more successful than use of LNG-IUD alone. Pronin et al. [38] conducted a study on women with early-stage EC by using LNG-IUD with injection of GnRHa; 72% of the patients recruited reached complete remission; 8 patients of the study developed 10 pregnancies in total with 8 live births. Dhar et al. [39] evaluated the effectiveness efficacy of LNG-IUD and GnRHa on 90 patients with low-grade EC; 68 patients (75.5%) reached complete response, and among 68 patients, 34 patients were prepared for pregnancy—3 of these patients became pregnant naturally and 13 conceived successfully with ART protocols. These positive results on LNG-IUD + GnRHa open a scenery on the effective, usefulness and necessity of progesterone oral administration for conservative treatment. 

### 2.3. Metformin in Fertility Sparing

There is evidence that suggests that diseases associated with insulin resistance are also a risk factor for EC. It has been reported that patients with BMI >25 kg/m^2^ usually have a higher risk for EC developing [40] and a bad effect on response rates of conservative treatment, highlighting the importance of obesity as a cause of EC, and the maintenance of normal BMI during conservative treatment to not invalidate its effectiveness [28,41]. Metformin is a biguanide worldwide used as a first-step therapy for type 2 diabetes, which inhibits hepatic glucose output and intestinal glucose adsorption, and promote the uptakes of glucose by skeletal muscle to alleviate insulin resistance. Mallik et al. [42] demonstrated that metformin has also been suggested to be a potential anticancer agent. Many studies established the role of metformin as an inhibitor of many kinds of tumors like pancreatic, medullary thyroid, breast, prostate, ovarian and endometrial carcinoma, in a dose-dependent manner via altering glucose metabolism, inhibiting the PI3K-AKT-mTOR signaling pathway, and promoting the apoptosis of cancer cells [43]. Obese patients have excessive adipose tissue in which biosynthesis of estrogen may occur. Greater amount of adipose tissue also means higher levels of adipokines and inflammatory factors strictly linked with the carcinogen process that led to EC [44,45]. Furthermore, type 2 diabetes is another risk factor for EC as a high level of insulin is an independent factor for EC [46]. Mu et al. [47] conducted a study that suggested that insulin resistance serves a central role in the pathogenesis of EC, and as insulin sensitizer, metformin improves the utilization of insulin by body tissues to reduce insulin resistance. As a result, serum insulin decreases and, consequently, EC risk decreases. Additionally, Yates et al. [48] reported that metformin increase adiponectin gene expression levels in obese patients, promote the secretion of adiponectin and induce apoptosis of EC cells. Yang et al. [49] conducted a prospective randomized controlled trial to investigate the efficacy of metformin plus MA compared with MA alone as fertility-sparing therapy for women with atypical hyperplasia or EEC. They concluded that the early complete response rate might be improved by adding metformin into MA therapy also for women without obesity or other components of metabolic syndrome. Mitsuhashi et al. [50] retrospectively analyzed long-term outcomes of MPA plus metformin as a fertile-sparing treatment for atypical hyperplasia and EEC, demonstrating that adding metformin to MPA led to a complete response in 81% of cases with only 10% of recurrence rate. MPA plus metformin achieved a high response rate, low recurrence rate and good fertility outcomes for fertility-sparing treatment of atypical hyperplasia and EEC, especially if the patient was obese. Thus, it is logical thinking that metformin may improve the early complete response rate of progestin therapy for fertility sparing in early-stage EC or atypical hyperplasia. Data on the use of metformin are encouraging, unfortunately, they are not strong enough to support metformin plus progestin treatment as a clinical routine, and more studies are needed to make these data more meaningful.

### 2.4. Surgical Treatment by Hysteroscopy

Although recent studies [33] showed that hysteroscopy followed by progestin therapy has the best outcomes in terms of complete response, the role of hysteroscopy as integrative therapy for fertility-sparing treatment in EC is still not well defined because most of the studies related to the use of hysteroscopy are clinic cases. A systematic review [51] of 39 years of published studies of young patients affected by early stage and low grade of EC with unsatisfied motherhood desire, treated with hysteroscopic resection, shows that the complete response rate was 88.9%. Pregnancy rate was 22% which increases to 66% if ART is applied. It suggests the potential role of hysteroscopy against the hormone treatment alone. Mazzon et al. [52] reported a series of patients treated with hysteroscopic resection of tumor, adjacent endometrium and under myometrium, and oral MPA (400 mg/daily) or MA (160 mg/daily) started 5 days after the hysteroscopy. Pregnancy rate for these patients was high also without ART (65%) and they were able to conceive also after 24 months after the end of the therapy. Additionally, studies regarding hysteroscopic treatment followed by LNG-IUD are extremely encouraging. Laurelli et al. [53] described that 78% of the patients with early stage of EC recruited for the study, and treated with hysteroscopy followed by LNG-IUD, reached the complete response to the therapy and only 7% of patients had a cancer recurrence; 45% of them after complete response removed the IUD and were able to conceive. Giampaolini et al. [54] demonstrated that hysteroscopic treatment followed by LNG-IUD has a high efficacy as fertility-sparing treatment, and strongly recommended this kind of approach since the insertion of LNG-IUD should have a lower relapse rate than progestin therapy alone, with similar response and pregnancy rate. However, many studies [55,56] suggest that hysteroscopy may influence the obstetrics outcomes due to mechanical damage of the endometrium causing Asherman’s syndrome and raising the risk of placental accretism. Data on the use of hysteroscopy with progestin or LNG-IUD are encouraging, and often gave better outcomes than hormone therapy alone. Unfortunately, they are not strong enough to support hysteroscopy plus hormone treatment as a clinical routine, and more studies are needed to make these data more meaningful.

### 2.5. Follow-Up

Patients who have chosen fertility sparing have to understand that a strict follow-up to assess the response is necessary. Complete remission for patients undergoing fertility-sparing treatment was described as 72% after 6 months and 78% after 12 months, suggesting that to have marginal benefits, hysteroscopy and imaging must be performed after 6 months and not before [29,56]. Current recommendations are for histological evaluation by endometrial biopsy through dilatation and curettage. In cases of complete response, conception must be encouraged. For women who want to delay pregnancy, even if controversial, maintenance treatment is mandatory. For patients who have satisfied their desire of motherhood, TH is highly recommended as definitive treatment [57], ovarian preservation could be considered depending on age and possible genetic mutations of the patient. Partial responders after 6 months of treatment may continue the treatment for another 3–6 months. If the disease is still confirmed by dilatation and curettage after the elongation of the conservative treatment, TH should be recommended [51,58].

### 2.6. Consideration about Psychological Impact

Gynecological chronic conditions could be heavily stressful for women [59]. The sexual function and psychological wellbeing of these women could be highly impaired after EC diagnosis. In particular, these women could be affected by depression, anxiety and impairment of quality of life [60]. Moreover, ART for fertility preservation strongly impacts on female psychological wellbeing. However, in cases of couple involvement, attention should be also given to the male counterpart [61]. Future perspectives about female psychological wellbeing should also be considered in EC management. 

### 2.7. New Molecules on Endometrial Cancer Supplementation

The majority of breast cancers and type-1 endometrial cancers show a similar overexpression of the estrogen receptors. To continue with, the risk of these types of tumors is higher in women who present elevated systemic estrogen levels [62]. Moreover, previous studies performed in these type of hormone sensible tumors suggest that insulin and insulin-like growth factors (IGFs) could have a role in endometrial tumor genesis. This action could be shared with estrogens [63]. As a consequence, aromatase inhibitors are molecules used in these hormonal sensitizing tumors since they have a negative effect on estrogen production. Moreover and more importantly, they have proved to be effective in reducing the long-term recurrence of estrogen-dependent cancer and the mortality rate [64]. However, a continuous treatment with aromatase inhibitors can be responsible for possible side effects. In particular, the continuous hypoestrogenic status can hamper women’s quality of life and, specifically, bone resorption. For this reason, new molecules have been studied which can supplement the aromatase inhibitor action. These molecules are part of natural compounds such as inositols. They are cyclic polyols with a main role in many metabolic pathways and are classified in nine stereo-isomeric compounds. Of them, the myo-inositol (MI) form is the most important and frequently found in nature [65]. In humans, inositols derive from the diet in the form of MI. Subsequently, MI is unidirectionally converted into different isomeric form such as D-chiro-inositol (DCI). This conversion is allowed thanks to insulin action and with the action of a specific Nicotinamide Adenine Dinucleotide (NAD)-NADH dependent epimerase [66].

**Figure 1:**
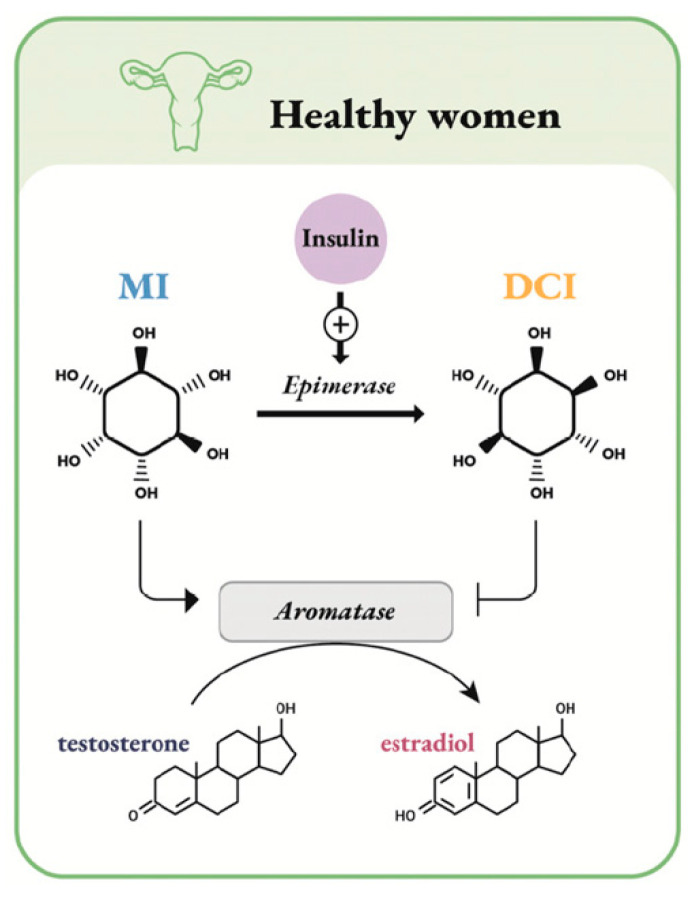
Schematic representation of inositol epimerase action. In healthy women, MI stimulates aromatase to produce estrogens, while DCI, obtained from insulin-dependent MI conversion by epimerase, has the opposite effect. Figure adapted from Unfer et al. [70].

Both MI and DCI are second messengers, after conversion, inside the cell as inositolphosphoglycans (IPGs) in their form as MI-IPG and DCI-IPG. These products both mediate the insulin signaling pathway but with some marked differences [67]. MI and DCI present different concentrations in tissues, suggesting different roles. These differences are detected addressing the MI/DCI ratio in tissues. The epimerase, which is responsible for the conversion of MI to DCI, has a tissue-specific activity [68] and has the key role for the MI:DCI specific ratios in different tissues and organs [69]. In this regard, Figure 1 shows the action of epimerase on MI to DCI conversion and its relationship with aromatase [70].

In particular, this ratio has been found around 40:1 in the peripheral blood [71], about 20:1 in the thecal cells, and about the range 70:1–100:1 in the follicular fluid of dominant follicles [66]. These ratios, as hypothesized, suggest a specific role for inositols, even though not defined yet. Of them, DCI supplementation has been found to have a key role in steroidogenic activity that can be distinguished in:

1. an indirect steroidogenic effect mediated by the insulin pathway;
2. an independent steroidogenic direct effect through downregulation of the aromatase gene expression and cytochrome P450 side-chain cleavage (P450scc) genes. These actions lead to an increase of testosterone synthesis.

DCI could synthetically act in the following ways. First, DCI causes a decrease in androgens in the short term followed by a decrease in estrogen [72]. Secondly, DCI focuses on aromatase and testosterone synthesis. This action causes androgen levels to increase once again [73]. However, when DCI is administered at higher doses, it can therefore act in the short-term period by increasing androgen levels [74]. For all these reasons, DCI can implement aromatase inhibitors’ activity by downregulating aromatase. Probably, DCI could act primarily as a supplement to established hormonal therapies, and further randomized controlled trials are needed in order to clarify its role. Similarly, these types of new molecules can act as a supplement in endometrial hyperplasia. This condition is defined as a pre-cancerous lesion of the endometrium. This lesion presents typically a high cell-proliferation rate that can result in endometrial carcinoma. Histopathologically and academically, endometrial hyperplasia can be divided into two forms: typical and atypical forms. In the first case, it can be seen as a single cell structure modification. In the second case, more than one cell structure modification is seen [75]. Among the risk factors, estrogens have a key role since they trigger endometrial proliferation in great proportion of cases. Moreover, an increased expression of aromatase P450 has been found in endometrial hyperplasia endometrium cells. This finding was peculiar for women with a history of polycystic ovary syndrome (PCOS) [76]. This overexpression has the potential to bring a local increase of estrogen levels with a subsequent mitogen stimulus. Aromatase inhibitors have been proved to play a main role in decreasing the local estrogen stimulus and in revealing atrophical endometrium [77]. However, considering the known properties of DCI, a potential role of this natural molecule can be found in treating endometrial hyperplasia. In fact, DCI acts in downregulating aromatase and, subsequently, reducing estrogen levels. Taking this into consideration, DCI may be an efficient supplement for reducing proliferation and treating the endometrial hyperplasia. As stated above, it is clear that well-designed RCTs are strongly needed, but the actual importance of inositols is day by day more evident and numerous targets of application are found [78,79,80].

### 2.8. The Molecular Role in EC

It is now elucidated that low-grade EECs and SECs have distinguishing molecular features. Type I EC, also known as endometrioid endometrial cancer, presents PTEN mutation, the most frequent somatic mutation. Moreover, this mutation commonly co-occurs with PIK3CA and PIK3R1 mutations in human EECs. In addition, a co-operativity has been proved between Pten loss and Ctnnb1 (which encodes β-CATENIN) mutation or Mlh1 inactivation. On the contrary, serous endometrial cancers (SEC) present as first event of the mutation of p53. Moreover, the frequent occurrence of FBXW7, PIK3CA and PPP2R1A somatic mutations, as well as CCNE1 amplification have been proved to be associated with SEC carcinogenesis [81].

Nevertheless, epigenetic cell modifications are gaining importance in cancer etiopathogenesis and characterization. In particular, a group of molecules could be used as marker of clinical prognosis: the non-coding RNAs (ncRNAs). Moreover, ncRNAs can have also a diagnostic role. New insights propose a therapeutic marker role, helping in building an “epigenetic profile” [82]. Actually, various ncRNAs have been individualized as having a role in EC pathogenesis, and for which a functional characterization is available. Moreover, ncRNAs have been detected which have a functional interaction with the EC diagnostic and prognostic genes [83]. A panel actually still lacks, but its formulation seems undelayable for a more tailored target medicine, especially in rare but critical cases [84]. 

## 3. Conclusions

Young women with stage IA and low-grade, positive progesterone receptors EEC and non-metastatic involvement or risk factors may require conservative management for fertility sparing if the motherhood desire is not yet satisfied. Uterine preservation is a feasible option that also seems to be safe until conception. The management may be various. The use of oral hormone therapy, the LNG-IUD and the GnRHa, alone or combinate between each other, offer good results for fertility preservation. In the last years, the data on metformin combined with hormone therapy are extremely encouraging, especially for obese patients, but more data are needed. Hysteroscopic surgery plus progestin or LNG-IUD may reduce the recurrence rate and should be preferred to other treatments, thanks to this high remission rate, although deserving further studies. Therefore, the optimal treatment is still debated and no consensus has been reached yet. Guidelines for managing conservatively the fertility of these patients should be drawn up without ever forgetting that an individual approach for this problem is mandatory since each patient has different characteristics and expectations regarding motherhood.

## Figures and Tables

**Table 1:** The number of arrows indicates the strength of the association. ↑: Increased risk; ↓: Decreased risk; –: No evidence for an association; ?: Some evidence for association/lack of association but not confirmed; CCC: Clear cell cancer; E: Estrogen; END: Endometrioid cancer; HRT: Hormone replacement therapy; MUC: Mucinous cancer; P: Progesterone; SER: Serous cancer. Table adapted from Webb [5].

Risk Factor	Endometrial Cancer	Ovarian Cancer	Cervical Cancer
Medical History			
HPV infection			Necessary
Hysterectomy	↓↓↓	?	
Tubal sterilization		↓	
Endometriosis	–	↑ CCC and END	
Diabetes	↑		
Polycystic ovary syndrome	↑		
**Reproductive history and hormones**			
Older age at menarche	↓	–	
Younger age at menopause	↓	↓	
Parity	↓↓↓	↓↓↓	↑
Breastfeeding	–	↓	
Oral contraceptive pill	↓↓↓	↓↓↓	↑ (recent use)
HRT:			
–E only	↑↑↑	↑↑	
–Combined E + P	↓? (continuous)	↑	
Fertility drugs	–?	–?	–?
**Lifestyle**			
Overweight and obesity	↑↑↑	↑ (not SER?)	–
Physical activity	↓	?	
Diet	↑ Glycemic index	?	
Coffee	↓ coffee	–	
Tea	↓? green tea	↓? green tea	
Smoking	↓	↑↑ MUC	↑
Alcohol	–	–	
NSAIDs	↓?	?	
Talcum powder	–?	↑	

## Data Availability

All data are provided within this study.
